# Insights into Flood Risk Misperceptions of Homeowners in the Dutch River Delta

**DOI:** 10.1111/risa.13479

**Published:** 2020-04-20

**Authors:** Jantsje M. Mol, W. J. Wouter Botzen, Julia E. Blasch, Hans de Moel

**Affiliations:** ^1^ Institute for Environmental Studies (IVM) Vrije Universiteit Amsterdam Amsterdam The Netherlands; ^2^ Utrecht University School of Economics (USE) Utrecht University Utrecht The Netherlands; ^3^ Risk Management and Decision Processes Center The Wharton School University of Pennsylvania Philadelphia PA USA

**Keywords:** Affect heuristic, availability heuristic, flood preparedness, objective risk, risk perception

## Abstract

Flooding is one of the most significant natural disasters worldwide. Nevertheless, voluntary take‐up of individual damage reduction measures is low. A potential explanation is that flood risk perceptions of individual homeowners are below objective estimates of flood risk, which may imply that they underestimate the flood risk and the damage that can be avoided by damage reduction measures. The aim of this article is to assess possible flood risk misperceptions of floodplain residents in the Netherlands, and to offer insights into factors that are related with under‐ or overestimation of perceived flood risk. We analyzed survey data of 1,848 homeowners in the Dutch river delta and examine how perceptions of flood probability and damage relate to objective risk assessments, such as safety standards of dikes, as well as heuristics, including the availability heuristic and the affect heuristic. Results show that many Dutch floodplain inhabitants significantly overestimate the probability, but underestimate the maximum expected water level of a flood. We further observe that many respondents apply the availability heuristic.

## 1 INTRODUCTION

Flooding is one of the most significant natural disasters worldwide in terms of number of people evacuated and total economic damages (UNISDR, 2015). With both sea levels as well as population increasing in flood‐prone areas, the impacts of flooding are expected to increase further in the future (IPCC, 2012; Munich RE, 2018). Hence, it is becoming more important to implement flood damage reduction strategies. Recent evidence shows that damage reduction measures taken by private homeowners are cost‐effective and can substantially limit the expected damages from flooding (Kreibich, Bubeck, van Vliet, & De Moel, 2015). However, current voluntary investments in private flood damage reducing measures are low. A potential explanation is that flood risk perceptions of homeowners differ considerably from objective estimates (OEs), which may skew their assessment of the damage that can be avoided by risk reduction measures (Bubeck, Botzen, Kreibich, & Aerts, 2013; Siegrist & Gutscher, 2008). Flood risk perceptions further affect support for public investments in flood protection infrastructure (Ripberger et al., 2018). This leads to a growing interest in risk perception research, which is important for the design of effective risk communication campaigns that stimulate people to better prepare for increasing natural disaster risks (Botzen, 2013; Kellens, Terpstra, & De Maeyer, 2013).

The aim of this article is to assess possible flood risk misperceptions of floodplain residents in the Netherlands, and to offer insights into factors that are related with the under‐ or overestimation of perceived flood risk. We build upon previous studies that have examined flood risk perception in relation to knowledge of the causes of flood events (Botzen, Aerts, & van den Bergh, 2009), distance to a perceived flood zone (O'Neill, Brereton, Shahumyan, & Clinch, 2016), and climate change information (de Boer, Botzen, & Terpstra, 2016). However, a systematic assessment of flood risk misperceptions is lacking for the Netherlands, as well as more generally, as becomes evident from a comprehensive literature review on the topic of flood risk perception by Lechowska (2018). This study takes the analysis of flood risk misperceptions one step further by relating the type of misperception (over‐ vs. underestimation) to objective risk assessments, heuristics, and personal characteristics. Risk perceptions are an important component of theories of decision‐making under risk in both economics and psychology. This article examines drivers of risk perceptions from both domains to arrive at a comprehensive assessment of flood risk perceptions.

The Netherlands, with its long history of protection against potentially severe flooding, lends itself as a relevant case to study these relationships. Moreover, the Dutch government has released several informational campaigns,1See, e.g., www.onswater.nl and www.overstroomik.nl
 but flood risk perceptions have since not been evaluated. While respondents in our sample have not experienced a flood recently, we examine whether we find similar patterns of risk perception as in the sample of Botzen, Kunreuther, and Michel‐Kerjan (2015), where respondents recently survived a major hurricane.

This article is structured as follows: Section 2 presents our theoretical framework and hypotheses, Section 3 describes the methodology, Section 4 presents results, and Section 5 discusses these results in relation to the literature. Finally, Section 6 gives policy implications and concludes.

## 2 THEORY AND HYPOTHESES

In this section, we discuss several theories and motivate our hypotheses about specific relations between risk perceptions and explanatory variables. Risk perceptions are an important component of theories of decision under risk from both economics and psychology. In economics, theories of decision‐making under risk have taken rationality as a starting point. In psychology, the importance of intuitive thinking (System 1) has been stressed, which is defined as fast, automatic, and directed by emotional reactions, as compared to deliberative thinking (System 2), which requires more effort to undertake trade‐offs. Generally, individuals combine both modes of thinking and they may apply simple rules of thumb (heuristics) whenever the cost of deliberative thinking is perceived too high. Heuristics are quick and straightforward decision rules that can be used to deal with complex decision environments (such as flood preparedness decisions) without draining an individual's cognitive capacities (Tversky & Kahneman, 1973).

### 2.1 Objective Risk Assessment

An important economic model of individual decision‐making under risk is expected utility theory (EUT), which assumes that individuals assess the likelihood and consequences of several choice alternatives, and subsequently, choose the alternative that gives the highest expected utility (von Neumann and Morgenstern, 1947). When the objective likelihood is uncertain or unavailable, individuals may still maximize expected utility by using their own subjective estimates of probabilities and losses (Savage, 1954), which in our applications are the perceived flood probability and damage.

Kunreuther and Pauly (2004) postulated based on the expected utility framework that individuals facing low‐probability/high‐impact risks expect a low return from searching for information about their risk, and hence, are unlikely to be fully informed about the risk they face. This implies that perceptions of low‐probability/high‐impact risks are likely to be biased, but would still be related to the objective risk faced by individuals (Kunreuther & Pauly, 2004). This means that risk perceptions would at least partially relate to objective risk, and hence, the latter may relate to the degree to which people under‐ or overestimate their risk. Such a heterogeneity in risks is applicable to the Dutch flood risk context, because although flood probabilities are generally low, expected flood inundation depths vary considerable between areas. In line with EUT, we predict that individuals under higher actual flood risk have higher flood risk perceptions.

**Hypothesis 1a**. *Respondents who live in an area with a larger flood probability have higher flood risk perceptions than respondents living in an area with lower flood probability*.

Ruin, Gaillard, and Lutoff (2007) found that flash flood risk perception (expected damage) among French motorists was higher among those who lived close to the place of impact. In a similar study among Dutch homeowners, Botzen et al. (2009) found that individuals living close to a river have higher flood risk perceptions. Recent studies have confirmed these findings, both for expected probability (Lindell & Hwang, 2008; Miceli, Sotgiu, & Settanni, 2008) as for expected damage (O'Neill et al., 2016; Zhang et al., 2010).

**Hypothesis 1b**. *Respondents who live closer to dikes have higher flood risk perceptions than respondents who live further away from dikes*.

Generally, we expect that respondents who live in low‐lying areas have higher flood risk perceptions than those who live on higher grounds, simply because the houses of the latter cannot be reached by floods and because they will experience lower inundation depths if they are flooded.

**Hypothesis 1c**. *Respondents who live in low‐lying areas (as indicated by higher maximum water levels) have higher flood risk perceptions than respondents who live on higher grounds*.

### 2.2 Heuristics

A growing body of evidence shows that individuals often do not behave as if they were following EUT; they rather engage in intuitive thinking, using heuristics or simple rules of thumb to evaluate a certain situation (Kahneman, 2003; Slovic, Finucane, Peters, & MacGregor, 2004). These heuristics are potentially helpful in many situations in daily life, but systematic biases may occur when they are applied to low‐probability/high‐impact events, causing errors in risk judgments. This may lead to completely ignoring the risk as well as overreacting to a recent disaster (Kunreuther & Michel‐Kerjan, 2015). Several systematic biases have been documented in the flood risk domain: in particular, the affect heuristic (Keller, Siegrist, & Gutscher, 2006; Slovic et al., 2004) and the availability heuristic (Siegrist & Gutscher, 2006).

Loewenstein, Hsee, Weber, and Welch (2001) noted that affective feelings toward risk, such as worry, are important determinants of risk perception (affect heuristic). However, Sjöberg (2000) argued that it is crucial to distinguish between worry and hazard properties when analyzing risk perception. Sjöberg (2007) showed in three Swedish survey data sets (each *n >* 400) that negative emotions are the strongest predictors of perceived risk. Botzen et al. (2015) surveyed 1,035 floodplain residents in New York City and found that high levels of worry were related to a higher perceived flood probability.

**Hypothesis 2a**. *High degrees of worry about flooding are related to higher perceptions of the flood probability*.

When people lack objective information about a certain hazard, they might rely on local risk management. Previous research has found that individuals who distrust local risk management have higher risk perceptions of hazardous facilities, such as nuclear waste repositories (Slovic, Flynn, & Layman, 1991). Terpstra (2011) conducted three Internet surveys among 1,071 Dutch households vulnerable to flooding and found that individuals who trust local risk management expect the probability of a flood to be lower. Also, the survey by Botzen et al. (2015) revealed that high trust in flood risk management officials is related to lower anticipated flood damage. We thus expect that trust in flood risk management lowers perceptions of flood probability and damage.

**Hypothesis 2b**. *Individuals with a high level of trust in local flood risk management have lower perceptions of the flood probability and damage*.

A related cognitive bias is the availability heuristic, where the probability or frequency of events is judged to be higher when the event is easier to recall (Tversky & Kahneman, 1973). Generally, individuals overestimate the probability of an event if they have experienced it, and underestimate the probability of events they have not experienced before (Siegrist & Gutscher, 2006; Viscusi & Zeckhauser, 2006). A first‐hand flood experience may make the flood risk more salient and easier to recall, leading to higher subjective flood probabilities, which is reflected in lower housing prices (Bin & Landry, 2013) and higher insurance take‐up (Shao et al., 2017). Most empirical studies indeed find a positive relationship between flood experience and flood risk perception (Reynaud, Aubert, & Nguyen, 2013; Richert, Erdlenbruch, & Figuières, 2017; Royal & Walls, 2019), which gives us a rationale for the next hypothesis.

**Hypothesis 2c**. *Individuals with flood experience have higher perceptions of the flood probability*.

With the last severe coastal flood in the Netherlands dating back to 1953, we expect few respondents in our sample who personally experienced a flood in their homes. However, a larger group of respondents might recall high water levels in their neighborhood, for example, during the 1995 river floods, which could be an alternative indicator of the availability heuristic in the flood context. Działek et al. (2019) demonstrated that memory of flood events tends to decrease quickly over time, with individuals recalling significantly smaller flood surface areas two years after the initial survey. Media exposure could play a role in memorizing flood events, which could increase recall. Siegrist and Gutscher (2006) showed that media coverage can increase risk perceptions for individuals lacking personal experience with flooding. A recent empirical study confirmed that risk perception increases following media exposure of the 2013 tornado in Moore, Oklahoma (Zhao et al., 2019). Therefore, we expect a similar effect of recalling high water levels on flood risk perceptions as with the previous hypothesis concerning flood experience.

**Hypothesis 2d**. *Individuals who recall high water levels have higher perceptions of the flood probability*.

All in all, heuristics in the flood risk domain may lead to serious misperceptions. While there is a growing body of the literature on flood risk perceptions (cf. Kellens et al., 2013; Lechowska, 2018), few studies have examined the difference between individual risk perceptions and objective risk estimates with regard to natural hazards. One notable example is O'Neill et al. (2016), who examined the difference between real and perceived distance to a hazard source. They found that respondents who live in a flood zone but indicate that they are outside are generally higher educated and less worried about flooding. To the best of our knowledge, the only paper that examined the deviation between objective and subjective flood risk estimates with respect to both probability and damage is Botzen et al. (2015). The authors report substantial underestimations and overestimations for both aspects of flood risk, but, in general, respondents overestimate the flood probability and underestimate potential damage.

**Hypothesis 3**. *Individuals will overestimate the probability of a flood and underestimate the consequences (damage and water levels)*.

While Botzen et al. (2015) quantify flood risk misperceptions, and examine which variables relate to perceptions of the absolute level of the perceived flood probability and damage, they do not examine which variables contribute to under‐ versus overestimations of flood risk in particular. Therefore, we cannot motivate hypotheses about the variables related to misperceptions. Nevertheless, we will examine whether the variables we expect to influence flood risk perceptions also influence over‐ or underestimations of probability, damage, and water levels.

## 3 METHODOLOGY

### 3.1 Survey Method

We conducted a survey with a sample of 2,122 Dutch homeowners living in floodplains in May and June 2018. The Netherlands is a relevant geographical area for flood risk perception research, as it has a long history of protection against flooding. Approximately half of the country is located behind dikes, including the metropolitan area where the main business districts and the government are situated. These low‐lying areas (dike rings) are protected from flooding by large dike infrastructures, leading to one of the highest flood safety standards across the globe. For example, some dike rings at the coast have safety standards of 1:10,000, which means that the dikes are designed to withstand an extreme flood event that may occur once in 10,000 years. The consequences of flooding in this area could be catastrophic, with maximum potential damages of 100 billion Euros (Aerts, Sprong, & Bannink, 2008). Nevertheless, floodplain inhabitants might not be aware of the possibility of flooding, as the most recent severe river floods in the Netherlands occurred in 1993 and 1995 (even though none of the dikes breached), while the most recent coastal flood dates back to 1953.

**Fig 1:**
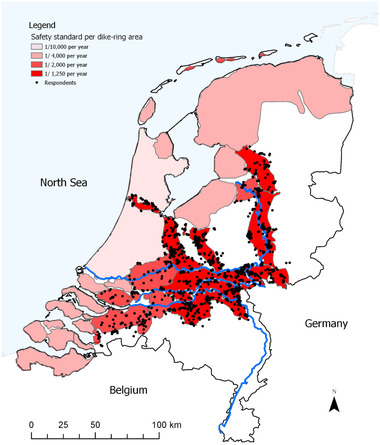
Locations of respondents to the survey on a map with safety standards of dike ring areas in the Netherlands. Every dot represents a respondent. Main rivers are indicated in blue.

We targeted homeowners in particular, as they bear the full costs of flood damage to their house, in contrast to tenants. We opted for an online survey instrument to reach a large sample of homeowners in flood‐prone areas. The invitation email did not specify the topic of the survey to prevent selection bias. The survey was distributed online and started with a selection question to ensure that only homeowners in predefined zip code areas could participate. Fig. 1 shows that respondents were located in the areas with relatively low dike ring safety standards (1:1,250 and 1:2,000 years, as opposed to 1:4,000 and 1:10,000 years in the coastal areas), in close proximity of the main rivers (Rhine and Meuse with their respective branches). The final response rate was 25.3%. We excluded 269 respondents who indicated that their home did not include the ground floor, which would give invalid results with respect to objective maximum water levels. From the 1,856 valid responses, 8 were incomplete, leaving 1,848 responses for analysis.

### 3.2 Elicitation of Dependent and Explanatory Variables

This section describes the questions of our dependent and explanatory variables, which were based on the previous surveys about disaster risk perceptions (Botzen et al., 2015; Bubeck et al., 2013). An extensive description of the survey, including a complete English translation of the questions, can be found in Mol, Botzen, and Blasch (2018).

Two questions were used to elicit respondents’ perception of the flood probability. Eliciting perceived flood probability estimates is a challenge, because individuals generally have difficulties with probabilistic concepts. In the context of influenza vaccination, which is a low‐probability/high‐impact event, analogous to flooding, Weinstein et al. (2007) showed that a qualitative question may better predict behavior under risk than a quantitative question on a percentage scale. Accordingly, we asked respondents about their perceived flood probability (*How large or small do you think the probability is that your house will be flooded?*) on a scale with seven answer categories. The drawback of such a question format is that people may attach different meanings to probability phrases, which complicates a comparison with objective, quantitative estimates.

**Fig 2:**
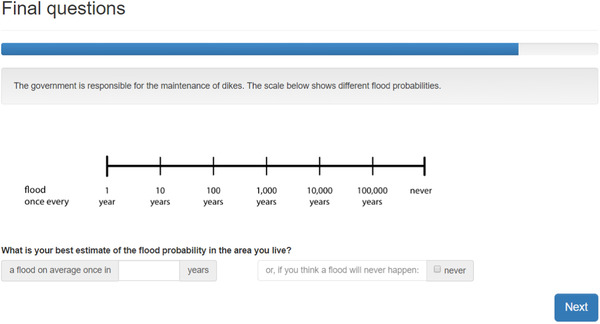
Decision screen of the subjective probability question, translated from Dutch. Respondents could either fill in an estimate on the left or tick the “never” box on the right, but not both.

To be able to quantify over‐ and underestimation among our respondents, we were interested in a more precise estimate of respondents’ perceived flood probability. Recent evidence shows that compared to percentage and frequency scales, a logarithmic scale performs best in eliciting low‐probability (<1%) perceptions in terms of validity, usability, and reliability (de Bruin, Parker, & Maurer, 2011; Woloshin et al., 2000). Therefore, we introduced a logarithmic scale with different return periods of flooding as a visual aid. Since our main interest is in flood probability misperceptions, we did not provide any anchor (compared to, e.g., Botzen et al., 2009, who used the legal safety norm as an anchor) with the scale. Fig. 2 shows the decision screen of this question. Respondents could either enter their best estimate of the flood probability or express their belief in a zero‐flood probability with the tick box on the right.

With regard to damage, we asked respondents to estimate potential flood damage to their house (*How much damage do you expect to your house and contents in case you would be flooded?*) on a scale with nine answer categories, as our pretest indicated that an open‐ended question would lead to substantial participant dropouts. An alternative indicator for perceived flood risk is the expected water level in a home once a flood occurs. We asked respondents about the water level during a flood, which might be easier to imagine and is therefore potentially less prone to errors. We used the following question: *Imagine your neighborhood is flooded, what height do you think the water would reach in your house?*, on a scale with six answer categories. We acknowledge that we asked for the *expected* water level in case of a flood, which is not identical to the *maximum* water level used as an objective indicator of flood risk. However, we believe that respondents who imagine a flood reaching their neighborhood will think about an extreme event, which may lead to answers corresponding to the maximum water level. In flood risk communication research, depicting maximum water level or inundation is standard (see, e.g., Lindner, Johnson, & Alsheimer, 2018). Moreover, communication about water levels by the Dutch government presents exactly these maximum water levels.2See www.overstroomik.nl. Lastly, there is little variance in flood water levels expected in the Netherlands due to the high safety standards, which result in either no flood (i.e., the dikes hold) or a large catastrophic flood (Vergouwe, 2015) with maximum or close to maximum water levels.

#### 3.2.1 Objective Flood Risk Indicators

The objective flood probability is equal to the legal return period of flooding as described in the 20093See https://wetten.overheid.nl/BWBR0025458/2016-07-01#BijlageI. Although a new water law was passed in 2017, the new law articulates that the flood protection infrastructure should meet the new norms only by 2050: https://www.helpdeskwater.nl/onderwerpen/waterveiligheid/primaire/nieuwe-normering/.
^,^ 4Sixteen respondents entered invalid letters in the zip code input field. We calculated their location based on the four digit zip code (PC4). Dutch water law, which was 1:1,250 for the majority (87%) of respondents, and 1:2,000 otherwise. Spatial information about objective flood risk was gathered with detailed geographical information system (GIS) maps of respondents’ zip codes (PC6).^4^ From these GIS maps, we calculated the distance to the nearest dike and the maximum objective water level for each respondent. The maximum objective water level was based on recent scenario estimates5
https://basisinformatie-overstromingen.nl/liwo/#/viewer/23
 provided by the Dutch government (Kok & Doef, 2008). Potential flood damage is typically estimated with depth‐damage curves, which provide the proportion of value at risk for a specific inundation depth (Merz, Kreibich, Schwarze, & Thieken, 2010). To obtain the approximate rebuilding value of the home, rather than the market value, we applied a standardization6Each home value was multiplied by the ratio of the average market price of the respective region and the average market price of the region with the lowest prices (Groningen). Data were obtained from: https://opendata.cbs.nl/statline/#/CBS/nl/dataset/83625NED/table?dl=19040
 to the continuous home values derived from the survey answers. We applied the damage curves of the Dutch SSM‐20177
https://www.helpdeskwater.nl/onderwerpen/applicaties-modellen/applicaties-per/aanleg-onderhoud/aanleg-onderhoud/schade-slachtoffer/
 of residential buildings to the rebuilding values, a fixed home content value of €70,000 and the maximum water level to calculate the objective damage per respondent (De Moel, Bouwer, & Aerts, 2014a).

#### 3.2.2 Heuristics

We asked several questions to elicit flood beliefs, based on the extensive reviews by Kellens et al. (2013) and Lechowska (2018). Kellens et al. (2013) classify frequently used variables in risk perception research into four main indicators: affect, awareness, likelihood, and impact. Note that the likelihood and impact (expected damage) variables have been discussed above in the dependent variables subsection. To measure affect (worry), we asked subjects to respond to a statement (*I am worried about the danger of flooding at my current residence*) on a five‐point Likert scale. We used the same linear coding for the statement on trust (*I am confident that the dikes in the Netherlands are maintained well*), which was almost an exact reproduction of the question in the original paper by Terpstra (2011). To assess previous flood risk experience, we asked respondents about damage (*Have you ever experienced damage to your house due to a flood?*). Furthermore, a Yes/No question was asked to examine recall of flood events (*Do you recall any situations of exceptionally high water levels in rivers close to your residence?*).

#### 3.2.3 Personal Characteristics (Control Variables)

Finally, personal characteristics such as gender, age, and numeracy may play a role in determining risk perceptions. We asked two questions about the probability of a certain weather in a respondent's residence, following Dillingh, Kooreman, and Potters (2016), to get a proxy for probability numeracy. Respondents who gave a larger estimate for “cloudy sky” than for “cloudy sky and rain” were coded as probability innumerate. Besides, risk preferences may be important when individuals evaluate risks (Loewenstein & Prelec, 1992): risk‐seeking individuals often foresee a lower probability of flooding (Botzen et al., 2009; Mills et al., 2016). We used a qualitative question to elicit risk preferences (*How willing or unwilling are you to take risks?*), as in Falk et al. (2018).

In the domain of natural hazards, sociodemographic variables such as education, income, and home value often explain little of the variance in risk perception (Peacock, Brody, & Highfield, 2005; Van der Linden, 2015). Considering the inconsistent effects of personal characteristics on risk perception in the previous literature (Kellens et al., 2013; Lechowska, 2018), we will adopt these variables as control variables in our analysis (see Table I for coding).

**Table I:** Summary Statistics

	*N*	Mean	*SD*	Min	Max
**Objective risk assessment**					
Sample area (0 = 1:1,250, 1 = 1:2,000)	1,848	0.13	0.34	0	1
Distance to nearest dike in km^a^	1,848	1.66	1.41	0.003	6.81
Maximum water level in m	1,848	1.34	1.37	0.00	8.29
**Heuristics**					
Worry about flooding^b^	1,848	2.08	0.96	1	5
Trust in dike maintenance^b^	1,848	3.88	0.83	1	5
Experienced flood damage (dummy)	1,848	0.06	0.24	0	1
Recall high water levels (dummy)	1,848	0.63	0.48	0	1
**Personal characteristics (control)**					
Gender (1 = female)	1,848	0.49	0.50	1	5
Age	1,848	53.76	14.49	18	90
Probability innumerate^c^ (dummy)	1,848	0.07	0.25	0	1
Risk aversion index^d^	1,848	4.49	2.04	0	10
Education^e^	1,848	5.86	1.43	1	9
Ln income^f^	1,389	7.95	0.42	5.52	8.57
Ln home value^g^	1,680	12.53	0.38	10.82	13.62

a Euclidian distance from center of zipcode area to nearest dike, based on GIS maps.

b Categorical answers, coded from 1 (strongly disagree) to 5 (strongly agree).

c Respondents were asked to estimate the probability of (1) a cloudy sky tomorrow and (2) a cloudy sky and rain. Respondents who gave a larger estimate for event (2) were counted as probability innumerate.

d How willing or unwilling you are to take risks? Categorical answers, coded from 1 (very unwilling) to 7 (very willing).

e Education in nine categories were: 1 indicates no diploma and 9 indicates a PhD.

f Respondents could indicate their after‐tax income category, starting at €0–€499, increasing in steps of €500. Continuous values of income variables were constructed by setting the income value of each respondent to the midpoint of the interval. €5,250 was used for the highest income category (*>*€5,000). The results were log‐transformed. Respondents who answered “Rather not say” or “Don't know” were excluded from this measure.

g Question format similar to income. Starting category *<*€100,000, increasing in steps of €50,000. €825,000 was used for the highest category (*>*€800,000).

### 3.3 Statistical Analysis

#### 3.3.1 Flood Risk Perceptions

We estimate various regression models where flood risk perception *Y* of individual *i* depends on a vector of objective risk variables (***O***), heuristics (***H***), and personal characteristics of the individual (***P***). The general specification takes the following form:

$$\begin{array}{ccc} Y{\left(\mathrm{flood}\;\mathrm{risk}\;\mathrm{perception}\right)}_{i} & = & \beta_{1}\;+\beta_{2}O_{i}+\;\beta_{1}H_{i}\\ & & +\;\beta_{1}P_{i}+\varepsilon_{i}, \end{array}$$

where *i* is the error term. In Model 1, the dependent variable *Y_i_* is a binary variable, indicating whether respondents answered “Zero” to the categorical flood probability question, which is why a probit model is employed as an estimation method. In Model 2, we use an ordered probit specification to estimate flood probability perceptions: the dependent variable *Y_i_* in this model is an ordinal variable that captures the categorical answer structure of the qualitative flood perception question. The dependent variable in Model 3 is the log‐transformed estimated flood probability (return period) and this model was estimated by ordinary least squares (OLS). Note that positive coefficient estimates indicate a high perceived flood probability in all three models. In Model 4, we estimate the perceived flood damage *Y_i_* with an ordered probit specification, to account for the categorical answer structure of the perceived flood damage question. We tested our data for multicollinearity, but this was not a concern: the correlation between the independent variables was small (*r <* 0.4).

#### 3.3.2 Flood Risk Misperceptions

To classify our respondents into those that underestimate, those that correctly estimate, and those that overestimate risk, we compared the perceived estimate (*PE*) of each respondent with the *OE*s, allowing for different error margins (*EM*s). The perceived risk estimate was considered correct if *OE*(1−*EM*) ≤ *PE* ≤ *OE*(1+*EM*). As an illustration, if the objective return period is 1:2,000 years and we allow for a 50% EM, we consider estimates under 1:3,000 years as underestimation and estimates above 1:1,000 years as overestimation, while estimates within that interval are correct. Since respondents were presented with fixed answer categories for the perceived damage and water‐level questions, we applied the EMs to the upper and lower bound of those intervals. For example, if a respondent answered “*10–50 cm*” for the perceived maximum water level, we considered this as correct if the OE was within the 5–75 cm interval (50% EM).

To understand the determinants of flood risk misperception in more detail, we estimated probit regressions where the dependent variable *Y_i_* is a dummy indicating underestimation (excluding overestimation) or overestimation (excluding underestimation) of individual *i*. The reference category in all models is the correct estimation.

### 3.4 Sample Characteristics

Our sample has equal proportions of male and female (49%) respondents. The average age of respondents is 54 years old and the distribution of age groups is very similar to that of homeowners in the general Dutch population.8
https://opendata.cbs.nl/statline/#/CBS/nl/dataset/83834NED/table?ts=1551260507456
 Ten percent have at least a master's degree as the highest education level, which is equal to the general population.9
https://opendata.cbs.nl/statline/#/CBS/nl/dataset/82816NED/table?ts=1551257782569
 The average after‐tax income category is €2,500–€2,999 per month, which corresponds to the average after‐tax income of the actual Dutch population (€2,933 per month, Netherlands Statistics, 2018a). The average home value of our respondents is €250,000–€299,000, which is slightly higher than the actual average home value in the Netherlands (€216,000, Netherlands Statistics, 2018b). Summary statistics of all explanatory variables used in the analyses are presented in Table I.

## 4 RESULTS

Flood risk is generally defined as the product of flood probability and flood damage. We first report respondents’ answers to the perceived probability, damage, and water‐level questions and relate them to the objective flood risk estimates. We analyze the drivers of flood risk perceptions in detail with a regression analysis to evaluate our hypotheses. Subsequently, we examine the direction of flood risk misperceptions by inspecting the predictors of under‐ and overestimations.

### 4.1 Flood Risk Perceptions

**Fig 3:**
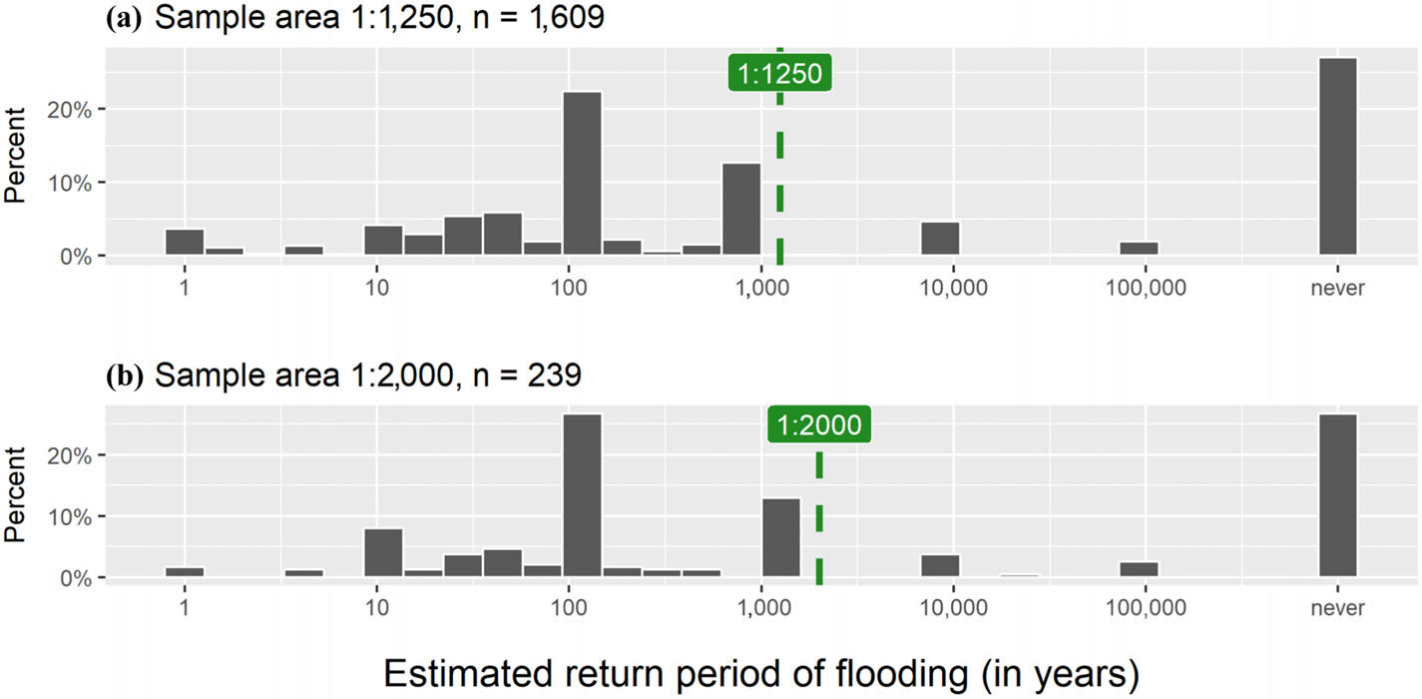
Histogram of respondents’ estimated return period of flooding. Green dashed reference lines indicate actual return periods.

**Fig A1:**
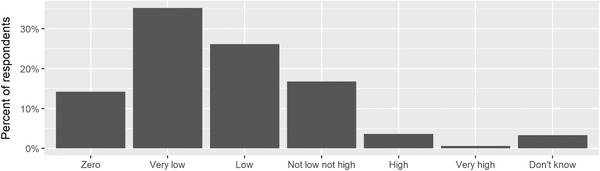
Histogram of respondents’ answers to the categorical flood probability question.

Few respondents (*<*5%) consider the probability of a flood as high or very high, which confirms that a large majority of Dutch citizens are aware of the high flood protection standards in the country. Almost 15% of respondents mark a perceived flood probability of zero in the categorical flood probability question (see Fig. A1 for the full distribution of answers). When asked to give a more precise estimate of the flood probability in the form of an estimated return period, more respondents report that a flood will never reach their current residence. Fig. 3 shows a histogram of the perceived return period of flooding, with dashed reference lines to indicate the objective return period. A large fraction of respondents (28%) expect that a flood will never occur at their present address, which is a serious misperception as the sample was drawn from the zip code areas that are at risk of flooding in the Netherlands (within dike ring areas with relatively protection norms). While these individuals may be unaware that they live in a flood‐prone area, other individuals largely overestimate the probability of a flood reaching their house. Approximately 10% of respondents estimate that the return period of a flood at their present address is 10 years or less, indicating a very high flood risk perception. Note that a return period of 100 years is considered a relatively high flood probability in the Netherlands, where most areas are protected up to 4,000 and even 10,000 years.

Overall, we find a bimodal pattern of risk perception, with a large group of respondents reporting high‐risk perceptions (return periods of 100 years and below) and a slightly smaller group who neglects the flood probability altogether. Very few responses were collected in between those two extremes.10To account for these different flood risk perception “types” in our data, we constructed a dummy variable to indicate the “never types.” We reran our regressions (not reported here) for this subgroup of “never types.” The sign and significance of the coefficients do not differ from the main regressions. When it comes to expected damage, the majority of respondents (70%) estimated that flood damage would cost up to €50,000.

#### 4.1.1 Objective Risk Assessment

Table II reports the results of our regression analyses. To examine the relationship between perceived and objective risk, consider the coefficients of the geographical characteristics in the first block of the table. We find no effect of objective return periods (as indicated by sample area) on the perceived probability of flooding, nor on perceived damage. In other words, the data do not support Hypothesis 1a. With regard to Hypothesis 1b, we find partial support. In Models 1, 3, and 4, we find no significant effect of dike distance on flood risk perceptions. The significantly negative coefficients of Model 2 indicate that respondents who live further away from dikes expect a lower probability of flooding than respondents who live closer to dikes, as hypothesized. We find, however, a significant, strong, and positive effect of the objective maximum water level on risk perceptions across all four models, confirming Hypothesis 1c.

**Table II:** Regression Results of Flood Risk Perceptions

	Probability	Probability	Probability	Damage
	*probit*	*oprobit*	*OLS*	*oprobit*
	(1)	(2)	(3)	(4)
Constant	−1.486		−10.926^**^	
	(1.561)		(4.214)	
**Objective risk assessment** Sample area (0 = 1:1,250, 1 = 1:2,000)	0.111	0.133	0.217	0.153
	(0.111)	(0.087)	(0.309)	(0.092)
Distance to nearest dike in km	0.014	−0.042^*^	−0.004	0.0003
	(0.029)	(0.021)	(0.075)	(0.023)
Maximum water level in m	0.115^***^	0.155^***^	0.288^***^	0.112^***^
	(0.032)	(0.023)	(0.079)	(0.025)
**Heuristics**				
Worry about flooding	0.444^***^	0.623^***^	1.443^***^	0.181^***^
	(0.055)	(0.043)	(0.121)	(0.034)
Trust in dike maintenance	0.048	−0.021	0.005	0.053
	(0.050)	(0.041)	(0.139)	(0.042)
Experienced flood damage (dummy)	0.268	0.675^***^	1.476^***^	−0.266^*^
	(0.254)	(0.140)	(0.431)	(0.119)
Recall high water levels (dummy)	0.408^***^	0.293^***^	1.183^***^	0.164^*^
	(0.083)	(0.064)	(0.245)	(0.074)
**Personal characteristics (control)**				
Gender (1 = female)	−0.060	0.121	0.099	0.088
	(0.085)	(0.064)	(0.232)	(0.072)
Age	−0.001	−0.009^***^	−0.014	−0.004
	(0.003)	(0.002)	(0.008)	(0.003)
Probability innumerate (dummy)	0.006	0.007	0.256	0.058
	(0.183)	(0.135)	(0.441)	(0.123)
Risk aversion index	0.068^***^	0.043^**^	0.131^*^	0.013
	(0.020)	(0.015)	(0.054)	(0.017)
Education	0.148^***^	0.073^**^	0.288^***^	−0.068^*^
	(0.033)	(0.023)	(0.087)	(0.027)
Ln income	−0.088	−0.157	−0.533	0.253^**^
	(0.100)	(0.083)	(0.275)	(0.096)
Ln home value	0.021	−0.040	0.168	0.506^***^
	(0.126)	(0.090)	(0.339)	(0.107)
Log likelihood	−668.8	−1,628.5	−3,816.9	−1,669.1
Pseudo *R* ^2^ (McFadden)	0.379	0.374		0.208
Observations	1,370	1,332	1,370	1,083
*R* ^2^			0.199	

*Notes*: Dependent variable Model 1: dummy estimated flood probability not zero; Model 2: categorical flood probability, higher numbers indicate higher flood probability; Model 3: log‐transformed estimated flood probability; Model 4: categorical damage estimate. Robust standard errors in parentheses.

^*^ *p <* 0.05;

^**^ *p <* 0.01;

^***^ *p <* 0.001.

#### 4.1.2 Heuristics

We find a strong effect of worry on flood risk perceptions across models. The significantly positive estimates for worry confirm Hypothesis 2a: individuals with high levels of worry about flooding estimate the likelihood of flooding to be higher. Moreover, the coefficient of Model 4 implies that those who worry a lot about flooding expect significantly higher damage to their house in case of a flood. We find no effect for trust in dike maintenance on flood risk perceptions: Hypothesis 2b cannot be confirmed. Individuals who have previous flooding experience, indicated by the dummy variable of “experienced flood damage” generally perceive a higher likelihood of flooding, as predicted by Hypothesis 2c. However, the results are not statistically significant in Model 1. Interestingly, individuals who have had their home damage due to flooding in the past have lower damage expectations for future floods. One explanation for this effect is that flood events in the Netherlands in the last decades have been relatively small, which may have led to minor damages. Finally, we find strong support for the use of the availability heuristic (Hypothesis 2d) in the data: individuals who remember high water levels have significantly higher flood probability perceptions for all three models.

#### 4.1.3 Personal Characteristics (Control Variables)

In addition to the explanatory variables related to our hypotheses, we observe some other interesting patterns with regard to our control variables. We find that respondents with a higher income generally expect higher damages. The significantly positive estimates for education indicate that more highly educated respondents perceive a higher likelihood of flooding, while the significantly negative estimate in Model 4 indicates that they expect a lower level of flood damage. Moreover, risk‐averse and younger respondents seem to have higher flood risk perceptions. We find no effect of gender and probability innumeracy on risk perceptions.

### 4.2 Flood Risk Misperceptions

**Fig 4:**
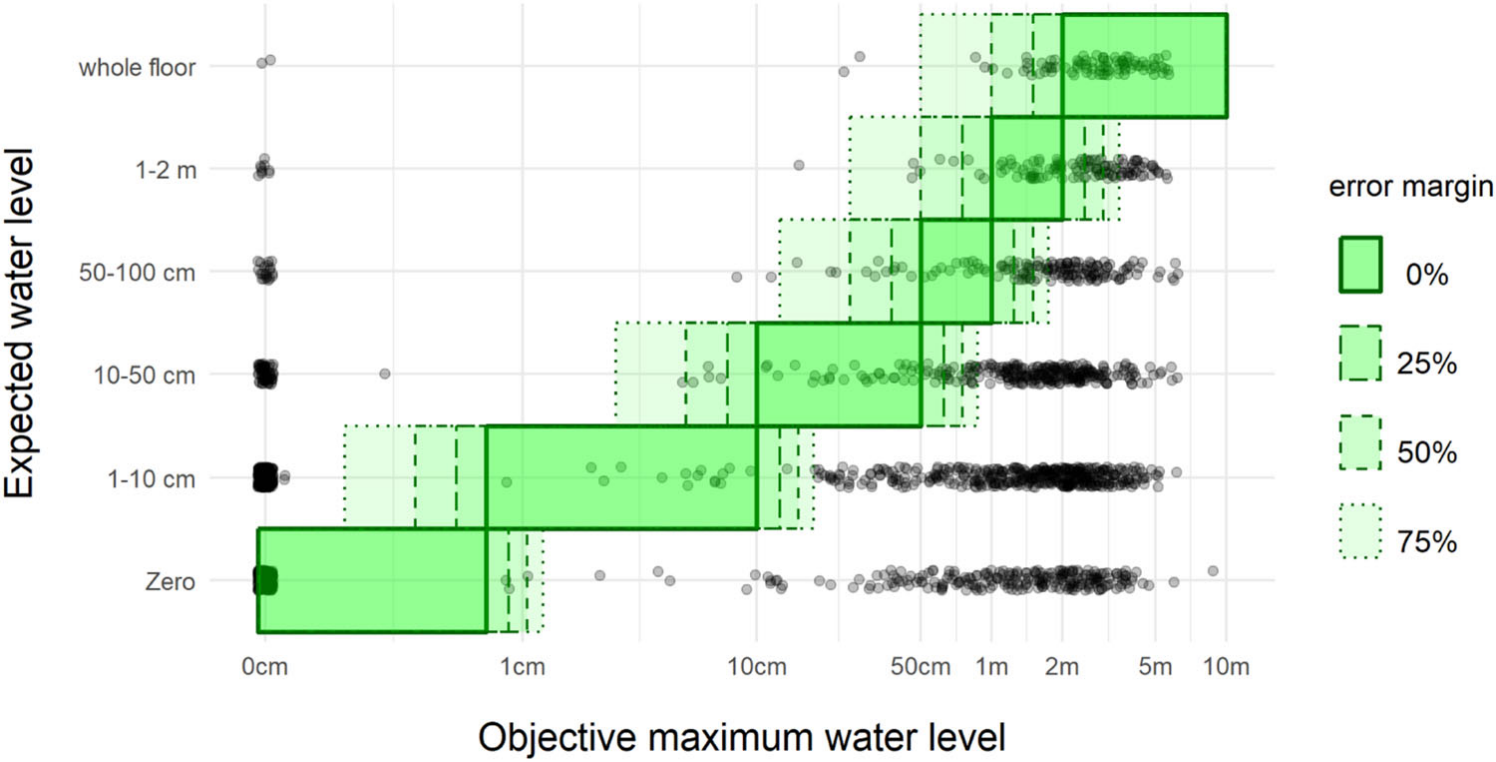
Perceived versus objective water levels; green shaded bars indicate correct estimates.

**Fig A2:**
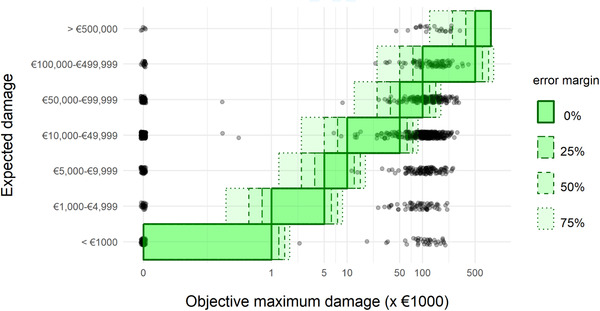
Perceived versus objective flood damage; green bars indicate correct estimates.

In this section, we examine the direction of flood risk misperceptions: over‐ versus underestimation. Fig. 4 shows a scatter plot of the perceived and the objective maximum water level. Each observation (respondent) is indicated with a gray dot with 1% random jitter to facilitate readability. The graph reveals a small subset of respondents who have zero as their objective maximum water level.11We have tested this subset on coding errors but none were found: these individuals simply live close to the border of a dike ring or on slightly higher grounds. For robustness, we reran our analysis on flood risk perceptions excluding this sample. The results do not change qualitatively. Green shaded bars indicate the range where perceived and objective water‐level estimates match. To acknowledge that flood risk involves large uncertainties and is therefore difficult to estimate for respondents, we allow for different EMs around the OE. All data points above the green diagonal represent respondents who overestimate maximum water levels, while data points below the diagonal represent those who underestimate. The graph shows that most Dutch homeowners seriously underestimate the maximum water level in their home in case of flooding, even when we allow for a 75% margin of error.12We use error margins following Botzen, Kunreuther, and Michel‐Kerjan (2015) and checked with experts whether the 25%, 50%, and 75% margins could be applied to the Dutch context. The reader is referred to De Moel, van Vliet, and Aerts (2014b) and Huizinga, Moel, and Szewczyk (2017) for a detailed discussion of uncertainty and sensitivity in flood risk modeling. A similar pattern emerges for the relationship between perceived and objective damage (see Fig. A2).

**Fig 5:**
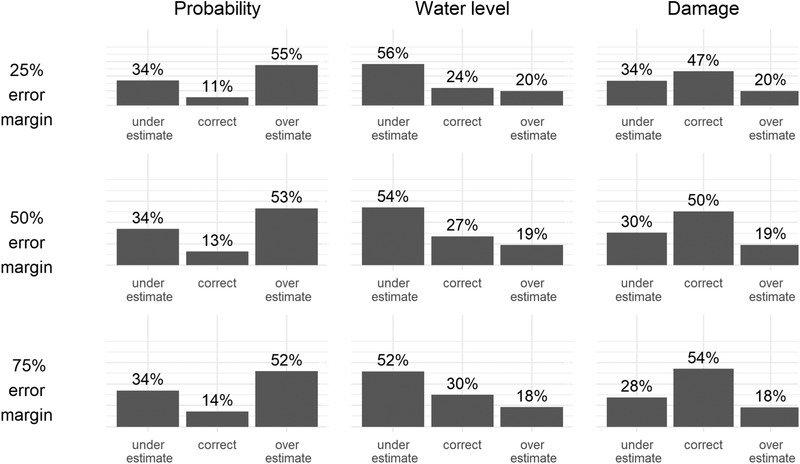
Distribution of flood risk perceptions at different error margins.

Fig. 5 gives an overview of the ratio of under‐, correct, and overestimations under different EMs for the three different aspects of flood risk perception (probability, water level, and damage). The majority of respondents overestimates the flood probability and underestimates the maximum water level, under all EM specifications, which is in line with Hypothesis 3. Fig. 5 also shows that respondents have more correct estimates when it comes to anticipated damage, rather than the maximum water level in case of flood.

Table III reports regression results of probit regressions on a dummy of underestimation versus correct estimation (excluding overestimation) or overestimation (excluding underestimation). The significantly positive constant term in Model 3 confirms that individuals generally underestimate the maximum water level during a flood, while the nonsignificant constant terms in Models 5 and 6 verify that most respondents correctly identify the expected flood damage.

**Table III:** Probit Regressions of Flood Risk Misperceptions

	Probability		Water level		Damage	
	Underestimate	Overestimate	Underestimate	Overestimate	Underestimate	Overestimate
	(1)	(2)	(3)	(4)	(5)	(6)
Constant	3.586	2.918	4.127^**^	1.513	−0.653	−1.328
	(2.094)	(1.752)	(1.556)	(2.164)	(1.526)	(1.746)
**Objective risk assessment**						
Sample area (0 = 1:1,250, 1 = 1:2,000)	−0.169	−0.007	−0.104	0.620^**^	−0.273^*^	0.205
	(0.174)	(0.146)	(0.139)	(0.195)	(0.128)	(0.157)
Distance to nearest dike in km	0.012	0.029	0.240^***^	0.027	0.153^***^	−0.008
	(0.044)	(0.034)	(0.034)	(0.049)	(0.029)	(0.038)
Maximum water level in m	−0.090	0.050	0.448^***^	−2.498^***^	0.389^***^	−0.762^***^
	(0.046)	(0.039)	(0.050)	(0.560)	(0.037)	(0.200)
**Heuristics**						
Worry about flooding	−0.230^***^	0.259^***^	−0.054	0.506^***^	−0.020	0.033
	(0.070)	(0.057)	(0.047)	(0.081)	(0.045)	(0.060)
Trust in dike maintenance	0.027	0.017	−0.102	−0.050	−0.121^*^	−0.079
	(0.074)	(0.065)	(0.054)	(0.074)	(0.049)	(0.059)
Experienced flood damage (dummy)	0.191	0.582^*^	0.136	0.199	0.150	0.581^*^
	(0.451)	(0.253)	(0.243)	(0.292)	(0.211)	(0.253)
Recall high water levels (dummy)	−0.408^***^	−0.027	−0.174^*^	0.022	0.065	0.073
	(0.119)	(0.114)	(0.085)	(0.125)	(0.089)	(0.096)
**Personal characteristics (control)**						
Gender (1 = female)	−0.057	0.031	0.097	0.182	−0.232^**^	−0.252^*^
	(0.119)	(0.106)	(0.093)	(0.136)	(0.088)	(0.111)
Age	−0.003	−0.008^*^	−0.011^**^	−0.013^**^	−0.008^*^	−0.011^**^
	(0.004)	(0.004)	(0.003)	(0.005)	(0.003)	(0.004)
Probability innumerate (dummy)	0.129	0.239	0.090	−0.025	−0.212	0.065
	(0.289)	(0.209)	(0.201)	(0.241)	(0.185)	(0.190)
Risk aversion index	−0.040	−0.006	−0.018	−0.032	0.033	0.006
	(0.026)	(0.023)	(0.021)	(0.029)	(0.019)	(0.023)
Education	−0.121^**^	−0.036	0.025	0.096	0.027	−0.062
	(0.044)	(0.042)	(0.034)	(0.050)	(0.032)	(0.038)
Ln income	0.126	−0.130	0.128	0.168	−0.026	0.180
	(0.153)	(0.153)	(0.108)	(0.182)	(0.112)	(0.133)
Ln home value	−0.183	−0.091	−0.371^**^	−0.258	0.043	0.092
	(0.169)	(0.145)	(0.130)	(0.178)	(0.129)	(0.153)
Log likelihood	−346.6	‐427.2	−573.2	−278.5	−639.4	−439.5
Pseudo‐*R* ^2^ (McFadden)	0.355	0.417	0.399	0.516	0.315	0.288
Observations	621	926	1,104	631	1,064	890

*Notes*: Probit regression estimates of misperception (over‐ and under‐) versus correct estimation (at 50% error margin) for three indicators of flood risk. Robust standard errors in parentheses.

^*^ *p <* 0.05;

^**^ *p <* 0.01;

^***^ *p <* 0.001.

#### 4.2.1 Objective Risk Assessment

The positive coefficients for the variable sample area indicate that respondents in the safer dike ring area are more likely to overestimate the maximum water level and less likely to underestimate the potential damage of a flood. The coefficients for dike distance indicate that individuals who live far away from dike protection significantly underestimate the maximum water level and the potential damage of a flood: “out of sight, out of mind.” The pattern of coefficients of maximum water level demonstrates that high‐risk individuals with high maximum water levels are more likely to underestimate water levels and damage. The pattern is consistent: these high‐risk individuals are also less likely to overestimate water levels and damage. We find no significant misperceptions of flood probability based on objective risk variables.

#### 4.2.2 Heuristics

Respondents with high levels of worry have serious overestimations of probability and water levels, but not of damage. High trust in dike maintenance makes it less likely that respondents will underestimate potential flood damage. This suggests that trust in dike maintenance does not activate a false sense of safety, which has raised concerns by previous researchers (see, e.g., Tobin, 1995, on the “levee effect”). Experience with flood damage increases the likelihood of overestimating flood probability and potential flood damage. Finally, we find that respondents who recalled high water levels are less likely to underestimate flood probability and maximum water levels.

#### 4.2.3 Personal Characteristics (Control Variables)

With regard to our control variables, we find that older individuals are less likely to have misperceptions (both under‐ and overestimations) on all three risk factors. The significantly negative estimate for education indicates that more highly educated individuals are less likely to underestimate the flood probability.13We conjectured that older participants would have more flood experience. Instead, we found a small but negative Pearson correlation between age and the experienced flood damage dummy (*ρ* = −0.081, *p <* 0.001) and that higher educated participants have more flood damage experience (*ρ* = 0.067, *p* = 0.004). We further found that younger people are more likely to feel worried about flooding (*ρ* = −0.160, *p <* 0.000), which may be one of the reasons why younger people have more misperceptions about flooding. However, education seems not to affect misperceptions about maximum water level and damage. Respondents with more expensive homes are significantly less likely to underestimate the maximum water level. We find no effects of risk aversion, income, and probability innumeracy on flood risk misperceptions.

## 5 DISCUSSION

This section discusses our main results in relation to our hypotheses and places these findings in the context of the existing literature. Starting with the indicators of objective flood risk, we find no support for the effect of flood probability (Hypothesis 1a) and dike distance (Hypothesis 1b) on flood risk perceptions. However, we sampled from two different protection standards, which were rather similar. This lack of initial variation could explain why our results do not show the hypothesized effect of flood probability on flood risk perceptions. We do find strong support for Hypothesis 1c: individuals living in low‐lying areas as indicated by maximum water level have higher subjective flood probability estimates, as well as higher potential flood damage estimates. The same individuals are more likely to underestimate water levels and damage. In other words, individuals living in low‐lying areas know that they face flood risks, but they underestimate them. One reason for the lack of effect of dike distance and the strong effect of maximum water levels is visibility. Respondents cannot easily observe the distance to the nearest dike, while maximum water level (which corresponds to the height of the land) may be easier to observe, for example, during periods of rainfall.

With regard to heuristics, we examined the affect heuristic, trust in dike maintenance, flood risk experience, and the availability heuristic. We find support for Hypothesis 2a: individuals with high levels of worry about flooding estimate the likelihood of a flood to be higher. These findings are consistent with Botzen et al. (2015), who find that low perceptions of flood probability are related to low worry and high trust in local flood risk management. However, the current analysis finds no support for Hypothesis 2b about the effect of trust in local flood risk management on flood risk perceptions. The lack of support for the trust hypothesis is in contrast to some previous work (Sjöberg, 2007; Terpstra, 2011) but not all (Carlton & Jacobson, 2013; Verlynde, Voltaire, & Chagnon, 2019). Moreover, trust in local flood risk management was rather high (less than 5% disagreed or strongly disagreed with the statement) in our sample.14We constructed a dummy variable for those who agreed or strongly agreed with the statement. We reran our analyses with this dummy variable. The sign and significance of the coefficients do not change. Future studies could examine the effect of trust on risk perception in a sample with more variability in trust ratings. Regarding Hypothesis 2c, note that only a small fraction of our sample has first‐hand flood experience (6%) and that we cannot exclude the possibility of reversed causality: individuals with higher risk perceptions are more likely to remember high water levels (cf. Osberghaus, 2017; Spence, Poortinga, Butler, & Pidgeon, 2011).

Indeed, we find ample support for Hypothesis 2d, which operationalized the availability heuristic as being able to recall a flood event. These findings are consistent with the previous findings on the effect of the availability heuristic on risk perceptions (Kellens et al., 2013; O'Neill et al., 2016).

Some limitations of our study should be addressed. First, the study uses an individualistic approach to risk perception, whereas homeowners might share their homes with family and discuss home‐related issues within their neighborhood. Van der Linden (2015) demonstrated that the behavior of others can be an important motivation to take action against flood risk. Future studies could examine the impact of social norms, an additional heuristic, on flood risk perception. Another limitation is that we used validated, but single‐item scales due to time constraints for respondents in completing the online survey. Some studies show that multiple‐item risk measures perform better in predicting risky behavior (Menkhoff & Sakha, 2017), but not all studies confirm this finding (Mol, Botzen, & Blasch, 2020). Numeracy and trust measures could be improved in future research by implementing a numeracy (McNaughton, Cavanaugh, Kripalani, Rothman, & Wallston, 2015) and trust (Grimmelikhuijsen & Knies, 2017) scale.

Our typology of flood risk misperceptions revealed that a majority of Dutch floodplain inhabitants overestimates the probability of a flood event, while underestimating the potential water level in case of a flood, supporting Hypothesis 3. Most damage estimates appear to be correct, although up to 34% of our sample underestimates potential flood damage. One explanation for this finding is that the maximum flood damage is bounded by the value of a home. Even without knowledge about depth–damage curves and water levels, respondents who opted for a certain fraction of the home value would have picked the right range quite often. These findings largely confirm the results of Botzen et al. (2015), who found that most New York City floodplain inhabitants overestimate flood probability, while underestimating the potential damage. A major difference between the two studies is that our sample has no recent flood experience, while the New York City sample was surveyed within one year after a major hurricane.

## 6 CONCLUSION

Flooding is one of the most significant natural disasters worldwide and its impacts are expected to increase further in the future. The implementation of damage reduction strategies is therefore of increasing importance. Damage reduction measures taken by private homeowners can be cost‐effective, but current take‐up is low. A potential explanation is that flood risk perceptions of individual homeowners differ considerably from OEs, which may alter their assessment of the cost‐effectiveness of damage reduction measures. Flood risk perceptions further affect support for public investments in flood protection infrastructure. While the literature on flood risk perceptions is extensive, so far a systematic assessment of the determinants of flood risk misperceptions was lacking. This article aimed to understand and quantify the flood risk misperceptions of Dutch floodplain residents, which is important for the design of effective risk communication campaigns and insurance schemes to cope with increasing natural disaster risks.

The main addition of this article to the literature lies in the detailed analysis of factors that are related with flood risk misperceptions. For instance, this analysis revealed that individuals who recall high water levels are less likely to have misperceptions of flood risk. It further shows that affective feelings about risk, in this case worry, may lead to overestimations of probability and water level. Experience of a flood and trust in dike maintenance seem to decrease flood risk misperceptions.

The following policy recommendations can be drawn from our results. The observation that a majority of respondents underestimate the water level of a flood implies that many Dutch homeowners may underestimate the cost‐effectiveness of damage reduction measures. It may hence be worthwhile for the Dutch government to proceed with information campaigns for homeowners in the river delta. The government could target homes that can be improved with cost‐effective measures. Moreover, these campaigns could specifically target homeowners in low‐lying areas as they are currently overrepresented in the share of underestimators of flood risk. A second implication of this study is that worry about flooding may increase flood risk perceptions, but it may lead to overestimations. Hence, a promising approach could be to focus on communicating consequential factors of risk, such as damage estimates and the maximum water level, as they are salient and rather easy to imagine, rather than communicating difficult to interpret probabilities or return periods. Future research could focus on the effectiveness of these informational campaigns, considering the absence of recent flood experience among Dutch floodplain inhabitants.

## Figures and Tables

**Fig B1:**
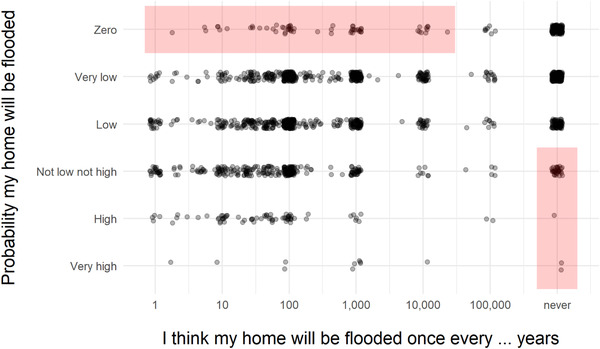
Categorical versus numerical flood risk perception; red shaded bars indicate respondents classified as inconsistent.

## APPENDIX A

### ADDITIONAL FIGURES AND TABLES

Fig. A1 shows a histogram of the given answers in the categorical question on perceived flood probability.

Fig. A2 shows a scatter plot of perceived flood damage and the objective flood damage. The figure confirms the pattern of Fig. 4; a large majority of respondents underestimate the damage that a flood can potentially cause.

## APPENDIX B

### INCONSISTENT TYPES

Since we used two different questions to elicit the perceived probability of a flood, we could examine respondents’ consistency. Fig. B1 shows a scatter plot of the categorical perceived flood probability versus the numerical estimate. We find a large variation in numerical estimates for the different probability phrases, which is in line with the previous research on interpretation of probability phrases (cf. Visschers et al., 2009; Willems et al.,^2019^). One could argue that the probability phrase *Very low* is inconsistent with a return period of 10 years. Our focus is on the most extreme answer categories, indicated with red bars in the figure: *Zero* on the categorical scale is clearly inconsistent with all numerical estimates *<*100,000 years and the explicit *never* answer to the estimated flood probability is inconsistent with all categorical estimates larger than *Low*. As a robustness check, we reran our analyses (not reported here) excluding inconsistent respondents (*n* = 97). All main effects and interactions remained unchanged.
